# Supplementary material for: An Ultrasensitive Room-Temperature H2 Sensor Based on a TiO2 Rutile–Anatase Homojunction
Source: Sensors (Basel). 2024 Feb 2;24(3):978. doi: 10.3390/s24030978 (PMC10856964; doi:10.3390/s24030978)
Supplement: Supplementary file 1 [file sensors-24-00978-s001.zip › sensors-2813897-supplementary.pdf]

Supporting information

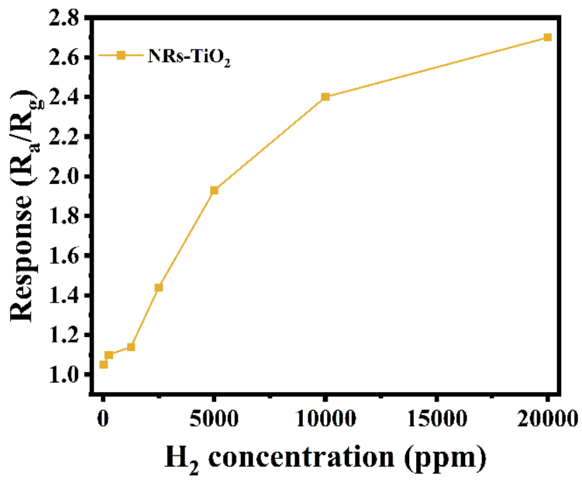

Figure S1 Response values of NRs-TiO$_2$ at different concentrations

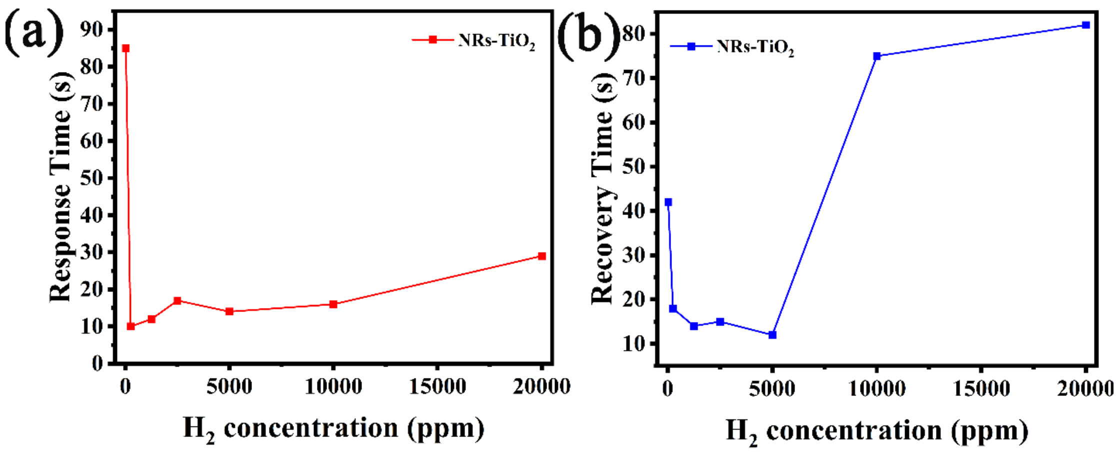

Figure S2 Response time (a) and recovery time (b) of NRs-TiO$_2$ at different concentrations
